# Job burnout and its impact on work ability in biosafety laboratory staff during the COVID-19 epidemic in Xinjiang

**DOI:** 10.1186/s12888-021-03555-x

**Published:** 2021-11-03

**Authors:** Yaoqin Lu, Qi Liu, Huan Yan, Sunyujie Gao, Tao Liu

**Affiliations:** 1 Department of Toxicology, School of Public Health, Xinjiang Medical University, Urumqi, 830011 Xinjiang China; 2 Urumqi Center for Disease Control and Prevention, Urumqi, 830026 Xinjiang China; 3 Department of Nutrition and Food Hygiene, College of Public Health, Xinjiang Medical University, Urumqi, 830011 Xinjiang China; 4 Xinjiang Engineering Technology Research Center for Green Processing of Nature Product Center, Xinjiang Autonomous Academy of Instrumental Analysis, Urumqi, Xinjiang China

**Keywords:** COVID-19, BSL staff, Job burnout, Work ability;

## Abstract

**Background:**

The coronavirus disease 2019 (COVID-19) has increased the physical and psychological stress of medical workers. This study was designed to investigate the prevalence and risk factors of job burnout and its impact on work ability among Biosafety Laboratory (BSL) staffs during the COVID-19 epidemic in Xinjiang.

**Methods:**

A total of 7911 qualified BSL staffs in Xinjiang were investigated by electronic questionnaires. The Maslach Burnout Inventory-General Survey (MBI-GS) was used for job burnout survey. Work Ability Index (WAI) was used for work ability survey. The prevalence and risk factors of job burnout in BSL staffs were analyzed through chi square test, t-test and one-way ANOVA. And then, the influence of demographic and job-related variables, i.e., confounding factors, were eliminated to the greatest extent by the propensity score analysis (PSA) method, to investigate the impact of job burnout on work ability in BSL staffs.

**Results:**

A total of 67.6% BSL staffs experienced job burnout. There were significant differences in the detection rate of job burnout among demographic and job-related variables, including gender, age, ethnicity, education, working years, professional title, marital status, number of night shift per month and overall sleep condition (all *P* < 0.05). The detection rate of job burnout in female was higher than that in male. The detection rates of job burnout in 45–50 years old, Han ethnicity, education of postgraduate or above, 11–20 years of working, intermediate professional title, married, staff with many night shifts per month and poor overall sleep condition were higher than that of other groups. The average burnout scores of the Emotional Exhaustion (EE), Cynicism (CY), Reduced Personal Accomplishment (PA) scale were 10.00 ± 5.99, 4.64 ± 4.59 and 15.25 ± 8.16, respectively. Multiple logistic regression analysis showed that the three dimensions of job burnout, i.e., EE, CY, PE, were negatively correlated with work ability and significantly affected the work ability of BSL staffs (all *P* < 0.001).

**Conclusions:**

Our results suggest that the prevalence of job burnout is extremely common among BSL staffs. In addition, the work ability decreases with the increase of job burnout and the improvement of job burnout can enhance work ability among BSL staffs.

## Background

The coronavirus disease 2019 (COVID-19) is a disease caused by a new type of coronavirus, which was named as Severe Acute Respiratory Syndrome Coronavirus type 2 (SARS-CoV-2) by Coronaviridae Study Group (CSG) of the International Committee on Taxonomy of Viruses [1]. On March 11, 2020, the World Health Organization (WHO) officially classified the global COVID-19 outbreak as a pandemic [2]. Due to the clinical characteristics of high infectivity, asymptomatic infection and early transmission of COVID-19 [3, 4], the number of infected people increased sharply. As of April 25, 2021, 146,054,107 confirmed COVID-19 cases and more than 3,092,410 deaths have been reported in 223 countries and regions around the world [5].

In the current pandemic, healthcare professionals (HCPs) are particularly vulnerable to emotional distress, as they work with factors such as risk of exposure to the virus, fear of infection, shortages of personal protective equipment (PPE), extended work schedules and the ongoing uncertainty of the pandemic’s evolution, which can have an impact on the mental health of them [6]. Numerous studies have shown that the COVID-19 pandemic significantly affected the mental health of HCPs. The Society of Critical Care Medicine surveyed 9492 intensive care unit clinicians in the U.S. and found that median self-reported stress, measured on a scale from 0 to 10, increased from 3 to 8 during the pandemic [7]. In addition, a study comparing the prevalence of burnout among HCPs during the pandemic with some studies conducted before the pandemic suggests that the rate of burnout among HCPs did increase during the epidemic in China [8]. With the explosive growth of the number of confirmed cases, rapid, effective, and accurate nucleic acid testing plays a key role in detecting suspected patients in a timely manner and blocking further spread of the virus. So far, molecular assays based on real-time polymerase chain reaction (PCR) to detect the SARS-CoV-2 genome are still considered as the gold standard for diagnosis of COVID-19. The biosafety laboratory (BSL) staffs, the implementers of diagnostic tests, will undertake a huge workload and endure long-term and high-intensity work during the COVID-19 epidemic. At the same time, they will inevitably confront a high risk of virus infection. According to statistics, as of April 3, 2020, about 10,000 medical staffs were infected by the virus and 74 of them died in Italy [9]. In addition, as of March 30, 2021, a total of 42 physicians have died of COVID-19 in Jordan [10]. BSL staffs, who play a key role in the fight against the pandemic as members of HCPs, are more vulnerable to burnout due to the greatly increased risk of contracting COVID-19 at workplace and the intense workload during a pandemic crisis.

Burnout is usually related to work, which is characterized by emotional exhaustion (EE), cynicism (CY), reduced personal accomplishment (PA). Due to the lack of personal resources to deal with the long-term pressure in work, people may easily suffer from burnout [11, 12]. In recent years, the issues of job burnout among medical workers have received more and more attention. A survey has reported that more than 50% of trained and licensed physicians suffer from job burnout in the United States [13]. In addition, according to research conducted by the Statistical Information Center of the Ministry of Health of China in 2010, 52.4% of health care professionals suffer from job burnout, and 3.1% of them meet high level criterion of burnout [14]. Moreover, a number of cross-sectional studies among medical workers have shown that job burnout is not only related to sleep disorders, depression, sedentary, obesity, musculoskeletal pain and many other health problems, but also related to the working ability [15, 16]. Because of the impact of job burnout, the probability of alcohol abuse among medical workers has increased by 25%, and the risk of suicidal ideation has doubled [17, 18]. Meanwhile, there has a decline in work efficiency and dissatisfaction at work, and the self-reported willingness to leave the current institution for reasons other than retirement has also doubled [19, 20]. Obviously, the syndrome of job burnout among medical workers can bring about a series of problems, such as high rate of medical error, high rate of medical malpractice claim, absenteeism and low work efficiency, and even can indirectly increase medical expenditure, which will seriously hinder the development of medical undertakings and reduce the ability of epidemic prevention and control. Relevant study has indicated that the incidence of burnout among medical workers working during the COVID-19 pandemic is higher than previously reported [21]. Therefore, we speculate that the outbreak of COVID-19 will further aggravate the degree of job burnout among BSL staffs.

Since the COVID-19 outbreak in November 2019, a large number of studies on the mental health of HCPs during the pandemic have focused on clinicians and nurses. This paper fills this gap in the literature by providing an in-depth exploration of the mental health of BSL staff during the COVID-19 pandemic [22, 23]. The results of testing by BSL staffs are a prerequisite and basis for the detection of suspected patients. In a pandemic situation, their work is even more necessary and critical, but their exposure to high risk of infection and intense workloads is also more pronounced. As of today, the global pandemic status seems to be far from over. Given the importance of BSL staffs in pandemic preparedness and control, this study aims to explore the job burnout and its impact on work capacity of BSL staffs in Xinjiang during the pandemic. This study aims to better understand this reality and to contribute as much as possible to this important group of BSL staffs in pandemic. The results not only could be used as the basis for the group intervention research of BSL staffs, but also provided scientific basis for further strengthening the nucleic acid detection capabilities during the COVID-19 pandemic.

## Methods

### Calculation of sample size for cross-sectional studies

The sample size formula for the present illness rate survey, $n=\frac{z_{\alpha /2}^2\times \mathrm{pq}}{\delta^2}$, p is the present-hazard rate, q = 1-p, 흳 is the tolerance error, generally taken as 0.1p, *z*_*α*/2_ is the significance test statistic, *z*_*α*/2_ =1.96 for α = 0.05, then the formula is calculated as, $n=400\times \frac{q}{p}$. According to the literature, more than 40% of nurses and more than 30% of radiographers and pharmacists suffered from job burnout during the COVID-19 pandemic in Japan [24]. In addition, a study of 2707 health workers from 60 countries showed that 51% of health workers had burnout syndrome [21]. In this study, we assumed a 30% prevalence of burnout to obtain the maximum required sample size. Which would calculate a sample size of 934, taking into account non-response and a 20% loss of questionnaires, which would require approximately 1167 people.

### Participants and procedure

This cross-sectional survey was conducted to assess the prevalence and risk factors of job burnout and its impact on work ability in BSL staffs from December 2020 to April 2021. In order to avoid face-to-face contact, the questionnaire star (a network tool for collecting questionnaire information) was used for online questionnaire survey. This study was highly representative. Seven thousand nine hundred eleven BSL staffs were recruited from 106 counties and districts of 4 prefecture level cities, 5 regions and 5 autonomous prefectures, involving all administrative regions and cities in Xinjiang. The inclusion criteria were as follows: (1) workers working in BSL in Xinjiang; (2) workers with a history of working in BSL for more than 1 year; (3) understand the purpose of this study and voluntarily participate in this survey.

### Measures

#### General investigation

The general investigation included ten demographic and job-related variables, including gender, age, ethnicity, education, working years, professional title, the level of BSL, marital status, number of night shift per month and overall sleep condition.

#### Job burnout investigation

The job burnout of this study was investigated using the Chinese version of the Maslach Burnout Inventory–General Survey (MBI-GS), which has been widely used in Chinese medical staff, and has been shown to have good reliability and validity [25]. The MBI-GS of Chinese version consists of three dimensions and 15 items: emotional exhaustion (EE, five items), cynicism (CY, four items) and reduced personal accomplishment (PA, six items). The score for each item is 7 points, ranging from 0 (never) to 6 (every day). Total score = 0.4 × Average score of EE + 0.3 × Average score of CY + 0.3 × Average score of PA. The total score was 0–1.49 for no burnout, 1.50–3.49 for mild to moderate burnout, and 3.50–6 for severe burnout [26].

#### Work ability investigation

This survey evaluated work ability in BSL staffs through the Work Ability Index (WAI) which not only has been widely used in the evaluation of work ability, but has been effectively verified in relevant studies in China [27, 28]. The questionnaire consists of seven parts (part, score range), which are the current work ability compared with the lifetime best (part 1, 0–10), work ability related to professional needs (part 2, 2–10), the number of current diseases diagnosed by doctors (part 3, 1–7), estimated work disabilities caused by diseases (part 4, 1–6), the sick leave in the past year (part 5, 1–5), the prediction of working ability in 2 years from now (part 6, 1,4 or 7), and mental resources (part 7, 1–4). The score of WAI was obtained by adding the scores of seven parts, which ranged from 7 to 49. The total scores of WAI were divided into four grades: poor (7–27), medium (28–36), good (37–43), and excellent (44–49). In order to facilitate the matching analysis of participants with PSA method, this study classified the good and excellent working ability as good (37–49 points), and the poor and medium working ability as poor (7–36 points).

#### The method of propensity score analysis

Propensity score analysis (PSA) was defined as the probability of exposure to events of interest given a set of baseline covariates [29]. Because it can minimize treatment selection bias [30], the method of PSA was widely used in the medical field especially in non-randomized and observational studies, and had gained more and more consensus [31, 32]. In this study, BSL staffs with different working abilities were compared by using the method of PSA which could minimize the selection bias and confounding differences among different working abilities.

### Quality control

Before the experiment, we conducted a pre-survey and provided informed consents explaining the purpose of the questionnaire and the principle of voluntary participation with the help of the person in charge of each BSLs. The trained investigators explained the purpose, significance, content and requirements of this study to all participants to ensure the quality and effectiveness of the online survey.

### Statistical analysis

The data collected were analyzed by R software 3.5.2 and SPSS 26.0. Percentages, means and standard deviations were the descriptive statistics used. Chi square test, analysis of variance (ANOVA) and independent t test were used to compare the differences of job burnout among demographic and job-related variables among BSL staffs in Xinjiang. In order to eliminate the demographic bias of participants, and to explore the impact of job burnout on their work ability among BSL staffs, the method of PSA was used to reduce the influence of demographic and work-related variables. After checking the data for multicollinearity and normality, the influence of three subscales in MBI-GS on WAI was analyzed by the method of multiple logistic regression. The significance level (α) set at 0.05.

## Results

### Characteristics

Baseline characteristics of participants are shown in Table 1. Among the 7911 voluntarily participating BSL staffs, more than half were female (76.20%), Han ethnicity (47.80%), over 30 years old (57.50%), married (59.40%), working more than 5 years (56.3%). In addition, 1481 (18.70%), 5671 (71.70%) and 759 (9.60%) of 7911 participates came from the first, second and third level of BSL respectively. In this survey, 5349 respondents met the burnout standard, and the prevalence rate was 67.61%. Chi square test showed that there were significant differences in sex, age, ethnicity, education, working years, professional title, marital status, number of night shift per month, and overall sleep between burnout group and non-burnout group (all *P* < 0.05). The job burnout detection rate of female, 45–50 years old, Han ethnicity, education level of graduate or above, 11–20 years of working, intermediate professional title, married, many evening shifts per month, and poor sleep condition were higher than that of other groups (all *P* < 0.05).

**Table 1:** Demographic data of participants with and without burnout (*N* = 7911)

Variables	Categories	Total (n = 7911)	Non-burnout (***n*** = 2562)	Burnout (***n*** = 5349)	Burnout detection rate (%)	Chi-squared value	***P*** value
Sex	Male	1882(23.80%)	655(25.60%)	1227(22.90%)	65.20%	6.594	**0.010**
	Female	6029(76.20%)	1907(74.40%)	4122(77.01%)	68.37%		
Age	20–25	1008(12.70%)	359(14.00%)	649(12.10%)	64.38%	50.904	**< 0.001**
	25–30	2358(29.80%)	870(34.00%)	1488(27.80%)	63.10%		
	30–45	3112(39.30%)	897(35.00%)	2215(41.40%)	71.18%		
	45–50	772(9.80%)	218(8.50%)	554(10.40%)	71.76%		
	≥50	661(8.40%)	218(8.50%)	443(8.30%)	67.02%		
Ethnicity	Han	3781(47.80%)	1143(44.60%)	2638(49.30%)	69.77%	18.580	**0.002**
	Uygur	2899(36.60%)	978(38.20%)	1921(35.90%)	66.26%		
	Kazak	590(7.50%)	205(8.00%)	385(7,20%)	65.25%		
	Hui	331(4.20%)	121(4.70%)	210(3.90%)	63.44%		
	Mongol	114(1.40%)	46(1.80%)	68(1.30%)	59.65%		
	Other	196(2.50%)	69(2.70%)	127(2.40%)	64.80%		
Education	Junior middle school	35(0.40%)	3(0.10%)	32(0.60%)	91.43%	12.446	**0.029**
	High school	29(0.40%)	9(0.40%)	20(0.40%)	68.97%		
	Secondary specialized school	1085(13.70%)	337(13.20%)	1085(13.70%)	68.94%		
	Junior college	3476(43.90%)	1149(44.80%)	2327(43.50%)	66.94%		
	undergraduate	3067(38.80%)	1002(39.10%)	2065(38.60%)	67.33%		
	Master or Doctoral degree	219(2.80%)	62(2.40%)	157(2.90%)	71.69%		
Working years	≤5	3410(43.10%)	1269(49.50%)	2141(40.00%)	62.79%	74.083	**< 0.001**
	6–10	1649(20.80%)	507(19.80%)	1142(21.30%)	69.25%		
	11–20	1340(16.90%)	353(13.80%)	987(18.50%)	73.66%		
	21–30	1038(13.10%)	281(11.00%)	757(13.10%)	72.93%		
	≥31	474(6.00%)	152(5.90%)	322(6.00%)	67.93%		
Professional title	Primary	6018(76.10%)	2076(81.00%)	3942(73.70%)	65.50%	52.664	**< 0.001**
	Middle	1300(16.40%)	324(12.60%)	976(18.20%)	75.08%		
	Deputy senior title	386(4.90%)	102(4.00%)	386(4.90%)	73.58%		
	Senior	207(2.60%)	60(2.30%)	207(2.60%)	71.01%		
The level of BSL	One	1481(18.70%)	457(17.80%)	1024(19.10%)	69.14%	2.150	0.341
	Two	5671(71.70%)	1851(72.20%)	3820(71.40%)	67.36%		
	Three	759(9.60%)	254(9.90%)	505(9.40%)	66.53%		
Marital status	Unmarried	2940(37.20%)	1075(42.00%)	1865(34.90%)	63.44%	44.592	**< 0.001**
	Married	4702(59.40%)	1394(54.40%)	3308(61.80%)	70.35%		
	Divorced	223(2.80%)	71(2.80%)	152(2.80%)	68.16%		
	Widowed	46(0.60%)	22(0.90%)	24(0.40%)	52.17%		
Number of night shift per month	Rarely	1306(16.50%)	514(20.10%)	792(14.80%)	60.64%	181.064	**< 0.001**
	Less	840(10.60%)	329(12.80%)	511(9.60%)	60.83%		
	General	3022(38.20%)	1084(42.30%)	1938(36.20%)	64.13%		
	More	2010(25.40%)	501(19.60%)	1509(28.20%)	75.07%		
	Many	733(9.30%)	134(5.20%)	599(11.20%)	81.72%		
Overall sleep condition	Very bad	890(11.30%)	134(5.20%)	756(14.10%)	84.94%	442.531	**< 0.001**
	Poor	1626(20.60%)	363(14.20%)	1263(23.60%)	77.68%		
	General	4006(50.60%)	1353(52.80%)	2653(49.60%)	66.23%		
	Better	1027(130%)	496(19.40%)	531(9.90%)	51.70%		
	Well	362(4.60%)	216(8.40%)	146(2.70%)	40.33%		

### Score analysis of three subscales of MBI-GS for BSL staffs

The MBI-GS subscale scores grouped according to demographic and job-related variables were shown in Table 2. The job burnout of BSL staffs in this survey was evaluated by MBI-GS, in which EE represents psychological feeling dimension, CY represents interpersonal dimension, and PA represents self-evaluation dimension in job burnout. The average burnout score of the EE scale was 10.00 ± 5.99, the CY scale was 4.64 ± 4.59, and the PE scale was 15.25 ± 8.16.

**Table 2:** MBI-GS subscale scores in demographic and job-related variables (*N* = 7911)

Variable	EE	CY	PA
Sex			
Male	6.30 ± 0.15	4.77 ± 0.11	8.75 ± 0.20
Female	5.89 ± 0.08	4.53 ± 0.06	7.96 ± 0.10
T	−1.229	0.122	−2.916
P	0.219	0.902	**< 0.001**
Age			
20–25	8.37 ± 5.36	3.83 ± 4.13	16.29 ± 8.79
25–30	9.21 ± 5.70	4.20 ± 4.48	14.83 ± 8.49
30–45	10.63 ± 6.07	5.08 ± 4.73	15.41 ± 7.85
45–50	10.92 ± 5.99	4.96 ± 4.44	15.56 ± 7.36
≥ 50	11.31 ± 6.58	4.98 ± 4.80	14.03 ± 8.04
F	51.307	22.360	9.920
P	**< 0.001**	**< 0.001**	**< 0.001**
Ethnicity			
Han	10.85 ± 6.03	5.35 ± 4.59	14.19 ± 7.71
Uygur	9.15 ± 5.82	3.92 ± 4.53	16.49 ± 8.44
Kazak	8.86 ± 5.76	3.94 ± 4.46	16.60 ± 8.41
Hui	10.40 ± 6.18	4.54 ± 4.39	13.76 ± 7.96
Mongol	9.07 ± 6.38	3.75 ± 4.00	15.30 ± 9.37
Other	9.43 ± 5.21	4.23 ± 4.26	15.84 ± 8.06
F	33.126	37.248	32.545
P	**< 0.001**	**< 0.001**	**< 0.001**
Education			
Junior middle school	7.37 ± 4.91	3.66 ± 4.35	23.83 ± 7.75
High school	7.24 ± 4.26	4.28 ± 3.75	16.17 ± 8.66
Secondary specialized school	9.14 ± 5.94	4.21 ± 4.68	17.34 ± 8.53
Junior college	9.75 ± 5.96	4.43 ± 4.56	15.59 ± 8.18
undergraduate	10.57 ± 6.01	4.97 ± 4.59	14.06 ± 7.81
Master or Doctoral degree	11.04 ± 5.87	5.53 ± 4.25	14.53 ± 7.52
F	15.174	8.577	37.527
P	**< 0.001**	**< 0.001**	**< 0.001**
Working years			
≤ 5	8.82 ± 5.50	3.92 ± 4.20	15.13 ± 8.52
6–10	10.29 ± 6.02	5.00 ± 4.72	15.31 ± 8.04
11–20	11.32 ± 6.14	5.40 ± 4.72	15.50 ± 7.63
21–30	11.17 ± 6.28	5.28 ± 4.92	15.66 ± 7.88
≥ 31	11.23 ± 6.56	5.04 ± 4.89	14.34 ± 7.95
F	67.644	39.701	2.662
P	**< 0.001**	**< 0.001**	**0.031**
Professional title			
Primary	9.45 ± 5.79	4.31 ± 4.47	15.37 ± 8.30
Middle	11.61 ± 6.28	5.69 ± 4.92	15.38 ± 7.69
Deputy senior title	12.14 ± 6.07	5.59 ± 4.35	13.97 ± 7.18
Senior	11.98 ± 6.51	5.70 ± 4.89	13.29 ± 8.25
F	74.486	42.751	7.675
P	**< 0.001**	**< 0.001**	**< 0.001**
The level of BSL			
One	9.39 ± 5.78	4.62 ± 4.65	16.60 ± 8.29
Two	10.20 ± 6.01	4.64 ± 4.54	14.87 ± 8.04
Three	9.75 ± 6.13	4.70 ± 4.80	15.42 ± 8.53
F	11.370	0.093	26.630
P	**< 0.001**	0.911	**< 0.001**
Marital status			
Unmarried	8.93 ± 5.59	4.10 ± 4.33	15.12 ± 8.61
Married	10.66 ± 6.15	4.98 ± 4.72	15.35 ± 7.87
Divorced	10.40 ± 5.63	4.72 ± 4.38	14.96 ± 8.05
Widowed	9.22 ± 5.82	3.93 ± 4.52	14.63 ± 7.92
F	52.160	22.720	0.659
P	**< 0.001**	**< 0.001**	0.577
Number of night shift per month			
Rarely	8.32 ± 5.18	3.97 ± 4.01	15.21 ± 8.40
Less	8.88 ± 5.52	4.05 ± 4.19	14.52 ± 8.21
General	8.96 ± 5.41	4.12 ± 4.26	15.47 ± 8.27
More	11.56 ± 5.90	5.38 ± 4.73	15.51 ± 7.79
Many	14.29 ± 7.24	6.63 ± 5.88	14.55 ± 8.14
F	202.503	70.44	4.08
P	**< 0.001**	**< 0.001**	**0.003**
Overall sleep condition			
Very bad	14.94 ± 7.50	6.93 ± 5.72	15.37 ± 8.22
Poor	11.94 ± 5.67	5.68 ± 4.72	15.49 ± 7.60
General	9.16 ± 5.12	4.24 ± 4.22	15.49 ± 8.17
Better	7.35 ± 4.70	3.32 ± 3.73	14.28 ± 8.22
Well	6.04 ± 5.34	2.44 ± 3.73	13.93 ± 9.75
F	358.631	134.447	7.34
P	**< 0.001**	**< 0.001**	**< 0.001**

### Propensity score analysis

Table 3 shows the situations of participants before and after matching. Among the 7911 participants, 2735 were poor in working ability and 5176 were good in working ability. The PSA was used to minimize the following demographic and job-related confounding factors with a caliper value of 0. 02 and random seed of 1. A total of 10 general demographic and job-related variables were set as confounding factors, including sex, age, ethnicity, education, working years, professional title, the level of BSL, marital status, number of night shift per month and overall sleep condition. Finally, 2318 pairs of BSL staffs with similar demographic and work-related indicators were matched.

**Table 3:** Participants before and after matching (*N* = 7911)

Items	Control group	Positive group
Before matching	2735	5176
After matching	2318	2318
Unmatched	417	2858
Discarded	0	0

Table 4 shows differences among participants before and after PSA. Multiple logistic regression analysis was used to compare the factors related to work ability in BSL staffs before and after PSA, with work ability as the dependent variable and demographic and job-related variables as the independent variables. Before pairing, there were significant differences in whole demographic and job-related variables (*P* < 0.05). After PSA, there was no significant difference in other demographic and job-related variables except sex and ethnicity (*P* > 0.05).

**Table 4:** Comparison of propensity score matching for WAI (*N* = 7911)

Variable	***β*** (95% CI)	S.E.	Standard ***β***	OR (95% CI)	VIF	Wald	***P***-Value
Before matching (n = 7911, treated = 5176, control = 2735)							
Intercept	−0.16 (−0.67,0.34)	0.26	–	0.85 (0.51,1.41)	–	−0.63	0.529
Sex	−0.13 (−0.25, −0.01)	0.06	−0.12	0.88 (0.77,0.99)	1.02	−2.194	**0.028**
Age	−0.09 (−0.17, −0.01)	0.04	−0.19	0.91 (0.85,0.99)	2.92	−2.11	**0.035**
Ethnicity	−0.06 (−0.11, −0.02)	0.02	−0.14	0.94 (0.9,0.98)	1.03	−2.612	**0.009**
Education	0.25 (0.19,0.32)	0.03	0.42	1.28 (1.2,1.38)	1.11	7.377	**< 0.001**
Working years	−0.27 (− 0.33, − 0.2)	0.03	− 0.72	0.76 (0.72,0.82)	2.79	−8.045	**< 0.001**
Professional title	− 0.17 (− 0.25, − 0.08)	0.04	− 0.24	0.84 (0.78,0.92)	1.59	−3.724	**< 0.001**
The level of BSL	0.21 (0.11,0.31)	0.05	0.23	1.23 (1.12,1.36)	1.05	4.177	**< 0.001**
Marital status	−0.17 (− 0.28, − 0.05)	0.06	− 0.2	0.84 (0.75,0.95)	1.63	−2.81	**0.005**
Number of night shifts per month	−0.18 (− 0.22, − 0.13)	0.02	−0.44	0.84 (0.8,0.88)	1.17	−7.487	**< 0.001**
Overall sleep condition	0.58 (0.52,0.64)	0.03	1.17	1.79 (1.68,1.89)	1.08	19.609	**< 0.001**
After matching (n = 4636, treated = 2318, control = 2318)							
Intercept	−0.03 (− 0.6,0.55)	0.29	–	0.97 (0.55,1.73)	–	−0.092	0.926
Sex	−0.15 (− 0.29, − 0.01)	0.07	−0.13	0.86 (0.75,0.99)	1.02	−2.14	**0.032**
Age	0.02 (−0.07,0.11)	0.04	0.05	1.02 (0.94,1.12)	2.67	0.509	0.611
Ethnicity	0.06 (0.01,0.11)	0.03	0.14	1.06 (1.01,1.12)	1.03	2.415	**0.016**
Education	−0.05 (−0.12,0.03)	0.04	−0.08	0.95 (0.88,1.03)	1.11	−1.213	0.225
Working years	0 (−0.07,0.08)	0.04	0.01	1 (0.94,1.08)	2.57	0.123	0.902
Professional title	0.05 (−0.05,0.15)	0.05	0.07	1.05 (0.95,1.16)	1.52	0.993	0.321
The level of BSL	0.09 (−0.02,0.2)	0.05	0.1	1.09 (0.98,1.22)	1.05	1.634	0.102
Marital zstatus	−0.07 (−0.2,0.06)	0.07	−0.08	0.93 (0.82,1.06)	1.58	−1.054	0.292
Number of late shifts per month	0.01 (−0.04,0.07)	0.03	0.03	1.01 (0.96,1.07)	1.18	0.546	0.585
Overall sleep condition	0.05 (−0.01,0.12)	0.03	0.09	1.05 (0.99,1.12)	1.09	1.53	0.126

*β* Regression coefficient of logistics, *SE* Standard Error, *OR* Odds Ratio, *CI* Confidence Interval *VIF* Variance Inflation Factor, *Wald* Probability value of Wald Statistic, *P* Probability

### Multiple logistic regression analysis of the impact of job burnout on work ability

Table 5 shows that, the three subscales of MBI-GS: EE (OR 0.92, CI 95% 0.91–0.94), CY (OR 0.90, CI 95% 0.88–0.92) and PA (OR 0.92, CI 95% 0.91–0.93) were significantly associated with work ability among BSL staffs (*P* < 0.001).

**Table 5:** Effects of the job burnout on the work ability of BSL staff according to the multiple logistic regression analysis (*N* = 4636)

Variable	***β*** (CI95%)	S.E.	Standard ***β***	OR (CI95%)	VIF	Wald	***P***-Value
Intercept	2.76 (2.52,3)	0.12	–	15.8 (12.45,20.06)	–	22.697	< 0.001
Emotional exhaustion	− 0.08 (− 0.09, − 0.06)	0.01	− 0.93	0.92 (0.91,0.94)	1.69	− 9.578	**< 0.001**
Cynicism	−0.11 (− 0.13, − 0.09)	0.01	−1.05	0.9 (0.88,0.92)	1.55	−10.76	**< 0.001**
Reduced personal accomplishment	−0.08 (− 0.09, − 0.08)	0	− 1.38	0.92 (0.91,0.93)	1.13	− 18.286	**< 0.001**

*P* value < 0.05 indicates a statistical difference among groups, and the smaller the *P* value is, the more significant statistical difference

*β* Regression coefficient of logistics, *SE* Standard Error, *OR* Odds Ratio, *CI* Confidence Interval *VIF* Variance Inflation Factor, *Wald* Probability value of Wald Statistic, *P* Probability

### The validation of the stability of the results

To verify the stability of the results, we screened the sex of the 7911 participants into male and female groups, created model 2 (male group) and model 3 (female group), and conducted sensitivity analyses by comparing the results with the model 1 (whole population group). The PSM method was carried out separately for model 2 (male group) and model 3 (female group). Regarding of model 2, 494 pairs of male BSL staffs with similar demographic and work-related indicators were matched. Before pairing, there were significant differences in Education, Working years, Number of night shift per month and Overall sleep condition (*P* < 0.05). After PSA, there was no significant difference in other demographic and job-related variables (*P* > 0.05). Regarding of model 3, 1787 pairs of female BSL staffs with similar demographic and work-related indicators were matched. Before pairing, there were significant differences in in whole demographic and job-related variables except for ethnicity (P < 0.05). After PSA, there was no significant difference in other demographic and job-related variables except number of night shift per month and ethnicity (*P* > 0.05). We conducted multiple logistic regression analyses on males and females in turn to compare the results of the effect of job burnout on the work capacity of BSL staff. Table 6 shows that, the ORs of EE, CY and PA were basically in a range of values for all three models with *p*-values < 0.001, and we consider that the results that EE, CY and PA are influences on work ability are reliable.

**Table 6:** The validation of the stability of the results

Variable	OR (95%CI)			***P***-Value
	Model 1	Model 2	Model 3	
Emotional exhaustion	0.92 (0.91,0.94)	0.94 (0.91,0.97)	0.92 (0.90,0.94)	**< 0.001**
Cynicism	0.90 (0.88,0.92)	0.88 (0.84,0.92)	0.90 (0.88,0.92)	**< 0.001**
Reduced personal accomplishment	0.92 (0.91,0.93)	0.92 (0.90,0.94)	0.92 (0.91,0.93)	**< 0.001**

Model 1, whole population group, a total of 7911 participants including both male and female

Model 2, male group, a total of 1882 male participants

Model 3, female group, a total of 6029 female participants

*P* value < 0.05 indicates a statistical difference among groups, and the smaller the *P* value is, the more significant statistical difference

## Discussion

The COVID-19 outbreak was found in Wuhan, Hubei Province, China in December 2019 [33] and almost at the same time, the medical systems of various provinces and cities in China were urgently mobilized according to the pre-arranged plan of major epidemic. As an important part of medical workers, BSL staffs play an extremely important role in epidemic prevention. For example, the detection results of SARS-CoV-2 can be used to diagnose whether patients suffer from COVID-19, so as to further separate them from the population, and especially in the early stage of the outbreak, rapid and reliable testing of SARS-CoV-2 infected people will help to allocate limited medical resources more reasonably and decelerate the spread of the virus [34]. As the implementers of diagnostic tests, the huge workload and high-intensity work will undoubtedly have a negative impact on the mental health and work ability in BSL staffs during the epidemic period, which will reduce the effect of local epidemic prevention and control. Job burnout is considered to be particularly harmful to the social psychology of workers, and has a high incidence in the global population. In this study, we found that more than 67.6% of the BSL staffs met the criterion of job burnout, while a relevant study had showed that more than 40% of the nurses and more than 30% of the radiation technicians and pharmacists suffered from job burnout in Japan during the COVID-19 pandemic [24]. In addition, a research of 2707 medical workers from 60 countries showed that 51% of the medical workers had the syndrome of burnout [21].

Compared with other results in the literatures, the prevalence of job burnout among BSL staffs is higher in Xinjiang. The higher prevalence of job burnout among BSL staffs may be related to the following reasons. The first is the risk of exposure to viruses. In a 2002–2004 inspection of clinical laboratory directors, who participated in ClinMicroNet, an online forum sponsored by the American Society of Microbiology, approximately 33% of laboratories stated the incidence of at least one laboratory-acquired infections [35]. Therefore, the risk of exposure to the virus increases significantly when PCR testing is performed. Secondly, there is a fear of infection and infecting family members. In qualitative interviews with HCPs during the pandemic, the study found that fear of infection, shortage of health care workers and adequacy of personal protective equipment were the main factors affecting their physical and mental health and job satisfaction [10]. Moreover, a high risk of hospital transmission has been reported in some regions. For example, a cohort study in London showed that 44% of frontline HCPs from hospitals were infected with SARS-CoV-2 [36]. Then there is the worksheet for extended working hours. Based on the health considerations and the needs of epidemic prevention and control, nucleic acid testing was carried out regularly among residents during the normal prevention and control period, which will inevitably increase the workload and extend the working hours of BSL staffs. A study has also shown that working more than 8 h per day is associated with higher EE and DP scores compared to working 4–8 h per day and < 4 h per day [37]. In addition, two studies conducted in Yemen [38] in 2009 and Lebanon in 2010 [39] also showed a significant association between burnout and long working weeks. Finally, uncertainty about the evolution of the pandemic persisted. In the early stages of the outbreak, the little information known about the virus posed a serious challenge for health workers [40]. For example, the transmission route and potential pathogenicity of SARS-CoV-2 has not been clarified, exposing laboratory staff to a high risk of infection during testing [41]. Moreover, the global COVID-19 pandemic, which has claimed nearly five million lives, continues to rage around the world, and there is uncertainty about when the pandemic will end in the future [42].

On sex differences in the degree of burnout, a relevant study had found that the degree of burnout reported by female doctors was higher than that reported by male doctors [43]. In addition, in the dimensions of EE, CY and PA, some studies had demonstrated that men tend to score higher than women in the dimensions of EE and CY, and women were more likely to use social support to deal with job burnout than men [44]. However, this study showed that there was no difference between men and women in the two dimensions of EE and CY, and men reported a higher degree of PA than women. The difference may be related to the low proportion of men in BSL staffs in this survey (23.80%).

With regard to age and working years, we found that there were significant differences in the detection rate of burnout among different age groups and working years (*P* < 0.001) and with the increase of age and working years, the detection rate of burnout showed an increasing trend. A relevant study had shown that the risk of burnout symptoms of doctors under 55 years old was more than twice of that of doctors over 55 years old [45]. Moreover, age and working years also had significant differences in EE, CY and PA. Staffs over 30 years old and working more than 11 years were higher in EE and CY than other groups, but lower in PA. We speculated that this result may be associated with the fact that with the increase of staffs’ age and working years, their physical health conditions and physical functions had gradually declined, and then they were suffering from job burnout because of the heavy nucleic acid testing workload. At the same time, the groups of older and longer working years had more job experience and played a mainstay role in their job position, resulting in a higher sense of achievement, which explained the result that the score of this group’s PA was lower than that of other groups.

Regarding ethnicity and the level of BSL, there were significant differences in the degree of job burnout among different ethnic groups and the Han nationality had a higher detection rate of burnout. At the same time, different ethnic groups also had significant differences in the dimensions of EE, CY, and PA. In addition, different laboratory levels had significant differences in the dimensions of EE and PA. The differences in ethnicity and the level of BSL may be related to the larger number of Han nationality and second-level BSL among the participants. As for education and professional title, there were significant differences among them, and there were also significant differences in the dimensions of EE, CY and PE. We speculated that the higher the level of education and professional title was, the more important the role that they played in the BSL team would be, which pushed them to suffer from greater risk of job burnout.

The burnout detection rate of married people was higher than that of others. In addition, the scores of EE and CY in married people were higher than that of other people and there were significant differences in the dimensions of EE and CY. However, Shanafelt [46] found that having a partner or getting married would reduce the risk of burnout, and compared with doctors without peer or family support, their degree of burnout was lower, which were different from the results of this study. Furthermore, during the COVID-19 pandemic, a study also looked at the burnout levels of nearly 600 doctors working at Assiut University Hospital and found that single doctors were more likely to suffer from job burnout than married doctors in terms of marital status, and that they scored significantly higher on EE and DP, and lower on PA. And this study speculated that the result may be related to the support provided by partners [37]. The results of the above studies differ from those of the present study, which may be due to regional differences between the study participants. Firstly, there are geographical differences. The province of Xinjiang, located in Northwestern China, has a population of 24 million and borders 8 countries including Mongolia, Russia, Kazakhstan, Kyrgyzstan, Tajikistan, Afghanistan, Pakistan, and India. Due to its special geographical location, Xinjiang has a large population flow and a complex personnel situation, resulting in huge pressure on local government to prevent and control the epidemic situation. Based on the health considerations and the needs of epidemic prevention and control, the local people should be screened in a short time when the epidemic occurs, and nucleic acid testing was carried out regularly among residents during the normal prevention and control period. Due to the large number of people to be tested, BSL staffs are under enormous work pressure and it is inevitable that BSL staffs have to work frequent overtime and even night shifts. In addition, one study found that increased time spent with children and family during the pandemic was independently associated with a reduced risk of burnout [47]. Therefore, we think that the high rate of burnout among BSL staffs in Xinjiang may be related to the huge workload and the inability to spend time with their families properly. Finally, we speculated that the impact of the job burnout was huge on women, older, Han nationality, married, higher education level and higher professional title among BSL staffs during this COVID-19 pandemic.

Some related studies had reported that sleep was an essential part of life, and played an important role in human recovery and survival [48, 49]. Furthermore, sleep was not only closely related to human learning and memory, but also played a key role in emotion regulation and cardiovascular metabolism [50, 51]. This study showed that the burnout detection rate of the staffs who experienced many nights shift per month and poor overall sleep condition was higher than that of other groups (*P* < 0.001). Moreover, there were also significant differences in the dimensions of EE, CY and PA. Similar to the results of our study, a study [52] in French reported that increased frequency of monthly night shifts was associated with an increased risk of burnout. In addition, a recent study has found that sleep quality is associated with the onset of burnout and that good sleep quality helps nurses recover from work-induced fatigue and psychological stress [53]. Furthermore, a cross-sectional study conducted among Turkish nurses found that 79.1% of participants had poor sleep quality and that sleep quality was negatively associated with burnout [54]. Then, we speculate that night shifts and sleep deprivation may be potential causes of burnout and may contribute to suffer from burnout in susceptible individuals.

Multiple logistic regression analysis was used to determine three dimensions of MBI-GS, i.e., EE, CY and PA, were negatively correlated with work ability, and these three dimensions could predict lower work ability. This study found that the higher the burnout level of BSL staffs was, the lower their work ability would be. Therefore, we speculated that the improvement of job burnout level of BSL staffs could improve their work ability.

In conclusion, our study showed that there was a high rate of job burnout among BSL staffs in Xinjiang during the COVID-19 pandemic. Some demographic and job-related variables, including gender, age, ethnicity, education, working years, professional title, marital status, number of night shift per month and overall sleep condition are determinants of job burnout scores. In view of the syndrome of job burnout among BSL staffs in Xinjiang, it is necessary to take corresponding measures to alleviate this situation. At the organizational level, the concept of organizational resilience which has been proposed in many studies refers to building reserves prior to a crisis and establishing workplace cultures and systems, such as effective leadership and a culture of organizational justice, to buffer work stress and increase individual resilience [55, 56]. Based on this, we suggest that BSLs can address the following four areas to improve the burnout situation of BSL staffs. Firstly, in terms of the BSL’s organizational environment and management system. The BSL should ensure a reasonable workload and increase flexibility between staff home and work; a more standardized workflow with specific procedural documents for reference; and a fairer and more rational system of work organization with opportunities for staff career development and training. Secondly, regarding mental health of staffs, health education should be carried out regularly, and timely psychological consultation and guidance should be arranged; Then, in the aspect of health testing, BSLs can offer personal health monitoring for employees, such as mental health assessment and physical examination; Finally, in terms of work arrangement, the rest areas and rest days can be arranged for night shift employees to facilitate the physical adjustment of tired employees. At the individual level, measures can be taken in the areas of resilience, daily exercise and social support. On enhancing resilience, Since the introduction of Antonovsky’s salutogenesis as a basis for health promotion [57], and the Ottawa Charter for Health Promotion, the concept of resilience has stimulated extensive research. Resilience is changeable and can be improved through interventions. Moreover, HCPs are seen as one of the most important target groups for resilience interventions [58]. With regard to resilience, on an individual basis, the focus is on the prevention of possible stressors, possible reactions and symptoms, and the development of behavioral and cognitive coping strategies. Regarding of exercise routines. A clinical trial of the impact of conducting a 12-week motivational physical activity program on physician trainees showed an improvement in burnout scores compared to the control group [59]. Therefore, we recommend that BSL staffs be physically active and engage in activities such as yoga to alleviate severe burnout. On social support. Evidence suggests that health workers who have good relationships, both personally and professionally, are happier and at lower risk of burnout [60]. Therefore, it is particularly important to foster good family relationships and strong social networks at work and in life.

Our study has several limitations. First, a cross-sectional study cannot identify causal relationships among demographic and job-related variables, job burnout and work ability in BSL staffs and is prone to recall bias. In the future, intervention studies can be conducted enabling us to determine whether alleviating job burnout will improve work ability among BSL staffs. Secondly, it is not known whether these results can be extrapolated to other regions, other countries or other industries in China because the participants of this study are all BSL staffs in Xinjiang. Finally, not all potential influencing factors were included in our study.

## Conclusions

As we all know, BSL staffs with healthy psychology and excellent working ability play a crucial role in identifying suspected patients and curbing the spread of the epi-demic during COVID-19 pandemic. Our study found that there were significant differences in sex and ethnicity (*P* < 0.05), and the detection rate of burnout among female and Han was higher. In addition, there were also significant differences in age, working years, education and professional title (*P* < 0.05) and with the increase of age and working years, the detection rate of burnout was also higher. Furthermore, there was an interactive relationship between job burnout and work ability. Improving the degree of job burnout in BSL staffs could improve their work ability. Relevant BSL departments should take necessary measures to alleviate the job burnout of BSL staffs and improve their work ability.

## Data Availability

The datasets used during the current study are available from the corresponding author upon reasonable request.

## Abbreviations

COVID-19: The coronavirus disease 2019
BSL: Biosafety Laboratory
MBI-GS: Maslach Burnout Inventory-General Survey
WAI: Work Ability Index
PSA: Propensity score analysis
EE: Emotional Exhaustion
CY: Cynicism
PA: Reduced Personal Accomplishment
SARS-CoV-2: Severe Acute Respiratory Syndrome Coronavirus type 2
CSG: Coronaviridae Study Group
WHO: World Health Organization
PCR: Polymerase chain reaction
